# Copper-Targeted Therapy in Experimental Endometriosis: Effects of Ammonium Tetrathiomolybdate on Markers of the Interconnected Processes of Inflammation, Innervation, and Fibrogenesis

**DOI:** 10.3390/ijms27021099

**Published:** 2026-01-22

**Authors:** María Belén Delsouc, Rocío Ayelem Conforti, Ana Sofia Zabala, Verónica Palmira Filippa, Leonardo Mariño-Repizo, Sandra Silvina Vallcaneras, Marilina Casais

**Affiliations:** 1 Laboratorio de Biología de la Reproducción, Instituto Multidisciplinario de Investigaciones Biológicas de San Luis (IMIBIO-SL), CONICET, Facultad de Química, Bioquímica y Farmacia, Universidad Nacional de San Luis (UNSL), San Luis D5700HHW, Argentina; bdelsouc@gmail.com (M.B.D.); ro.conforti64@gmail.com (R.A.C.); asofia.zabala@gmail.com (A.S.Z.); ssvallcakrivo@gmail.com (S.S.V.); 2 Laboratorio de Histología, Facultad de Química, Bioquímica y Farmacia, Universidad Nacional de San Luis (UNSL), San Luis D5700HHW, Argentina; vpfilipp@gmail.com; 3 Instituto de Química de San Luis (INQUISAL), CONICET, San Luis D5700HHW, Argentina; lmarino@email.unsl.edu.ar

**Keywords:** endometriosis, copper, ammonium tetrathiomolybdate, inflammation, innervation, fibrogenesis

## Abstract

Endometriosis (EDT) is a chronic, estrogen-dependent disease characterized by inflammation, fibrosis, pelvic pain, and infertility. Current therapies show limited long-term efficacy and adverse effects, underscoring the need for novel therapeutic approaches. Elevated copper (Cu) levels have been reported in both patients and animal models of EDT, making Cu chelation a promising strategy. This work aimed to evaluate the impact of ammonium tetrathiomolybdate (TM) on the expression of markers related to the interconnected processes of inflammation, innervation, and fibrogenesis in mice with induced EDT. Twenty-four female C57BL/6 mice were assigned to Sham, EDT, or EDT+TM groups. Treatment with TM began on postoperative day 15, with samples collected one month after EDT induction. Peritoneal fluid cytokines (TNF-α, IL-1β, IL-6, TGF-β1) were quantified by ELISA. Endometriotic-like lesions were examined for mRNA expression of cytokines, neurotrophins (*Ngf*, *Bdnf*, *Ngfr*), neural markers (*Uchl1*, *Gap43*), neuropeptides and nociceptive markers (*Tac1/Tacr1*, *Calca/Calcrl/Ramp1*, *Trpv1*), and fibrogenic markers (*Vim*, *Acta2*, *Col1a1*, *Fmod*) by RT-qPCR. Neurotrophin protein levels were measured by ELISA, and collagen content was assessed through Masson’s staining. TM significantly modulated inflammatory, neural, nociceptive, and fibrogenic markers, reducing most of them along with collagen content. These findings suggest that TM could impact key pathological mechanisms involved in EDT.

## 1. Introduction

Endometriosis (EDT) is a chronic, estrogen-dependent inflammatory disease in which endometrial-like tissue grows ectopically outside the uterine cavity, leading to fibrosis and pelvic adhesions. It is estimated that this condition affects approximately one in ten women of reproductive age and manifests with symptoms such as chronic pelvic pain, dysmenorrhea, dyspareunia, dysuria, and infertility [1].

Despite being an ancient disease, current therapeutic approaches, primarily hormonal or surgical, have a poor response. Therefore, there remains an unmet need for new, more effective, and less invasive therapies for this currently incurable condition [1,2].

Inflammation is a key process in the pathophysiology of EDT. Inflammatory cells, cytokines, and chemokines are found in the peritoneal fluid that sustains this inflammatory environment. Macrophages, mast cells, and neutrophils, along with other immune cells, are recruited to the endometriotic lesions, stimulating the production of factors such as tumor necrosis factor-alpha (TNF-α), interleukin-1 beta (IL-1β), interleukin-6 (IL-6), transforming growth factor-beta (TGF-β), neurotrophins, and various pain-related molecules. These mediators facilitate the recruitment of additional inflammatory cells, thereby generating a vicious cycle [3,4], which in turn promotes processes such as cell survival and proliferation, angiogenesis, neurogenesis, and tissue remodeling.

The aberrant innervation of peritoneal endometriotic lesions, characterized by a higher proportion of sensory nerve fibers compared to sympathetic fibers, is regulated by estrogens and neurotrophins [5]. Among the latter, nerve growth factor (NGF) and brain-derived neurotrophic factor (BDNF) are particularly relevant. Both increase the number of nerve fibers and promote the release of inflammatory mediators. In addition to these shared effects, NGF stimulates the expression of neuropeptides involved in pain transmission, such as substance P (SP, a member of the tachykinin family) and calcitonin gene-related peptide (CGRP). BDNF has been found to colocalize with macrophages and nerve fiber markers within endometriotic lesions, reinforcing its role in neuroinflammation and in the development of both inflammatory and neuropathic pain [3,6,7].

Fibrosis arises from the sustained interaction between inflammation and aberrant innervation, both of which converge on the activation of fibroblasts. In EDT, platelets, macrophages, ectopic endometrial cells, and sensory nerve fibers release cytokines such as TGF-β and neuropeptides. Together with hormonal signals, these factors drive the epithelial–mesenchymal transition (EMT) and pro-fibrotic signaling, promoting the persistence of endometriotic lesions [8,9]. In patients, fibrosis can contribute to the formation of adhesions, fertility problems, pain, and therapeutic resistance [10,11,12]; slowing its progression is essential.

Increased copper (Cu) levels have been reported in different studies in EDT and other gynecological pathologies [13,14,15,16]. This metal participates in cell proliferation, angiogenesis, cytokine secretion regulation, and the modulation of neurotrophic signals [16,17,18,19,20,21]. In this regard, Cu chelation could constitute a novel therapeutic strategy. Ammonium tetrathiomolybdate (TM) is a highly specific chelator with rapid absorption and a favorable safety profile. In a C57BL/6 mouse model of induced EDT, TM has previously been shown to decrease elevated Cu and estradiol levels and to reduce endometriotic-like lesion volume by affecting cell proliferation, angiogenesis, and redox imbalance [22,23]. In this context, this work aimed to evaluate the impact of TM on the expression of markers involved in the interconnected processes of inflammation, innervation, and fibrogenesis in mice with induced EDT. To our knowledge, our approach to restoring Cu homeostasis in this disease is pioneering, as is the exploration of the therapeutic potential of a chelating agent as a non-hormonal strategy.

## 2. Results

### 2.1. Effect of TM on Inflammatory Markers in Mice with Induced EDT

**Figure 1:**
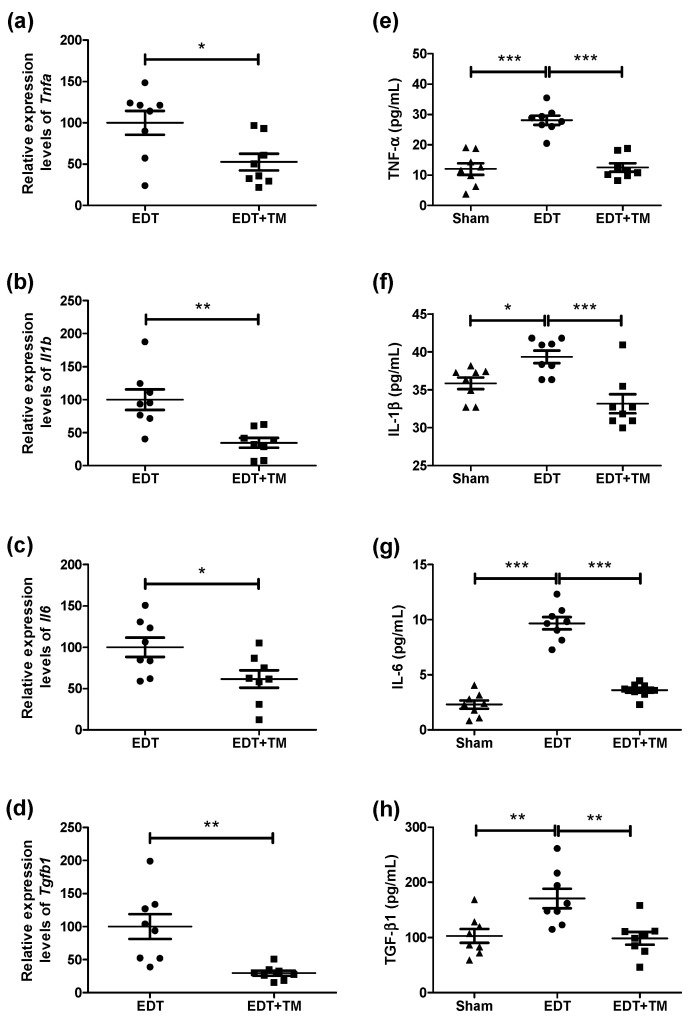
Effect of ammonium tetrathiomolybdate (TM) on inflammatory markers in mice with induced endometriosis (EDT). The mRNA expression of *Tnfa* (**a**), *Il1b* (**b**), *Il6* (**c**), and *Tgfb1* (**d**) in endometriotic-like lesions was evaluated by RT-qPCR in untreated mice with induced EDT or in TM-treated mice (EDT+TM). Relative quantification was calculated from the quantification cycle (Cq) values of the target genes and the reference gene (*Rn18s*) using the 2^−ΔΔCq^ method. Results are expressed as mean ± SEM (n = 8 animals per group). All experiments were performed in duplicate. Statistical comparisons were performed using Student’s *t*-test (* *p* < 0.05; ** *p* < 0.01). In addition, TNF-α (**e**), IL-1β (**f**), IL-6 (**g**), and TGF-β1 (**h**) levels were measured in peritoneal fluid by ELISA. Data are presented as mean ± SEM (n = 8 animals per group). One-way ANOVA followed by Tukey’s post hoc test was used (* *p* < 0.05; ** *p* < 0.01; *** *p* < 0.001).

Since inflammation is central to EDT and contributes to endometriotic lesion growth and associated symptoms, key cytokines were analyzed. Treatment with TM significantly reduced the mRNA expression of *Tnfa* (*p* < 0.05, Figure 1a), *Il1b* (*p* < 0.01, Figure 1b), *Il6* (*p* < 0.05, Figure 1c), and *Tgfb1* (*p* < 0.01, Figure 1d) in endometriotic-like lesions, compared to the EDT group. EDT induction in mice resulted in increased protein levels of TNF-α (*p* < 0.001, Figure 1e), IL-1β (*p* < 0.05, Figure 1f), IL-6 (*p* < 0.001, Figure 1g), and TGF-β1 (*p* < 0.01, Figure 1h) in peritoneal fluid. Furthermore, treatment with TM significantly reduced the concentrations of these cytokines compared to the EDT group, reaching values comparable to those observed in the Sham group (TNF-α, IL-1β, IL-6: *p* < 0.001; TGF-β1: *p* < 0.01, Figure 1e–h).

### 2.2. Effect of TM on the Innervation Process of Endometriotic-like Lesions

**Figure 2:**
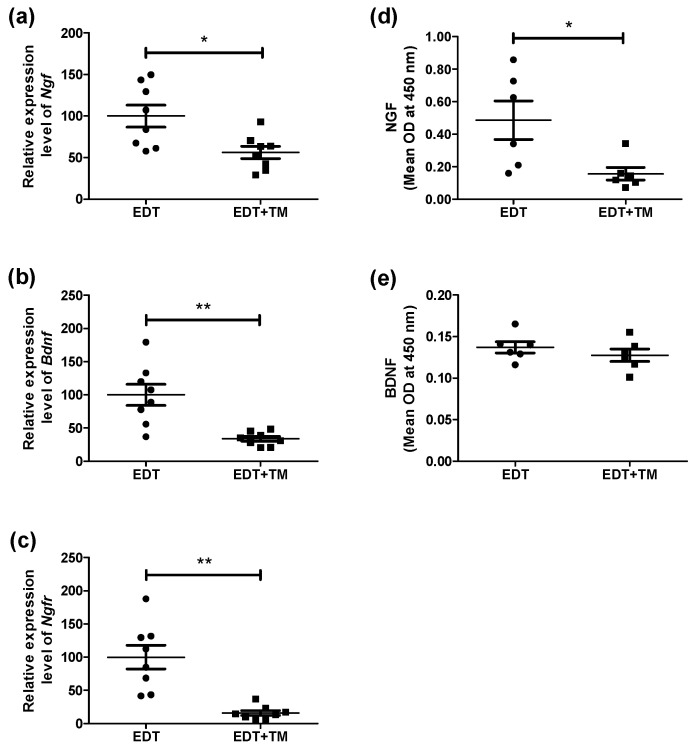
Effect of TM on the expression of neurotrophins and their common receptor in endometriotic-like lesions. The mRNA expression of *Ngf* (**a**), *Bdnf* (**b**), and *Ngfr* (**c**) was evaluated by RT-qPCR in untreated mice with induced EDT or in TM-treated mice (EDT+TM). The relative quantification of each mRNA was calculated from the Cq values obtained for the target genes and the reference gene (*Rn18s*), using the 2^−ΔΔCq^ method (n = 8 animals per group). ELISA assessed the protein levels of NGF (**d**) and BDNF (**e**) in both experimental groups (n = 6 animals per group). Results are expressed as mean ± SEM. All experiments were performed in duplicate. Statistical comparisons were carried out using Student’s *t*-test (* *p* < 0.05; ** *p* < 0.01). OD: optical density.

Inflammation and nerve growth in endometriotic lesions are deeply linked and contribute to pain modulation. Because neurotrophins promote nerve growth, we analyzed whether TM treatment could affect NGF and BDNF expression in the endometriotic-like lesions. First, it was observed that, in the group of animals treated with the Cu chelator, the mRNA expression of *Ngf* (*p* < 0.05), *Bdnf* (*p* < 0.01), and their common receptor *Ngfr* (*p* < 0.01) was significantly reduced compared to the EDT group (Figure 2a–c). Additionally, TM treatment significantly reduced NGF protein levels (*p* < 0.05, Figure 2d), whereas BDNF remained unchanged compared to the EDT group (Figure 2e).

**Figure 3:**
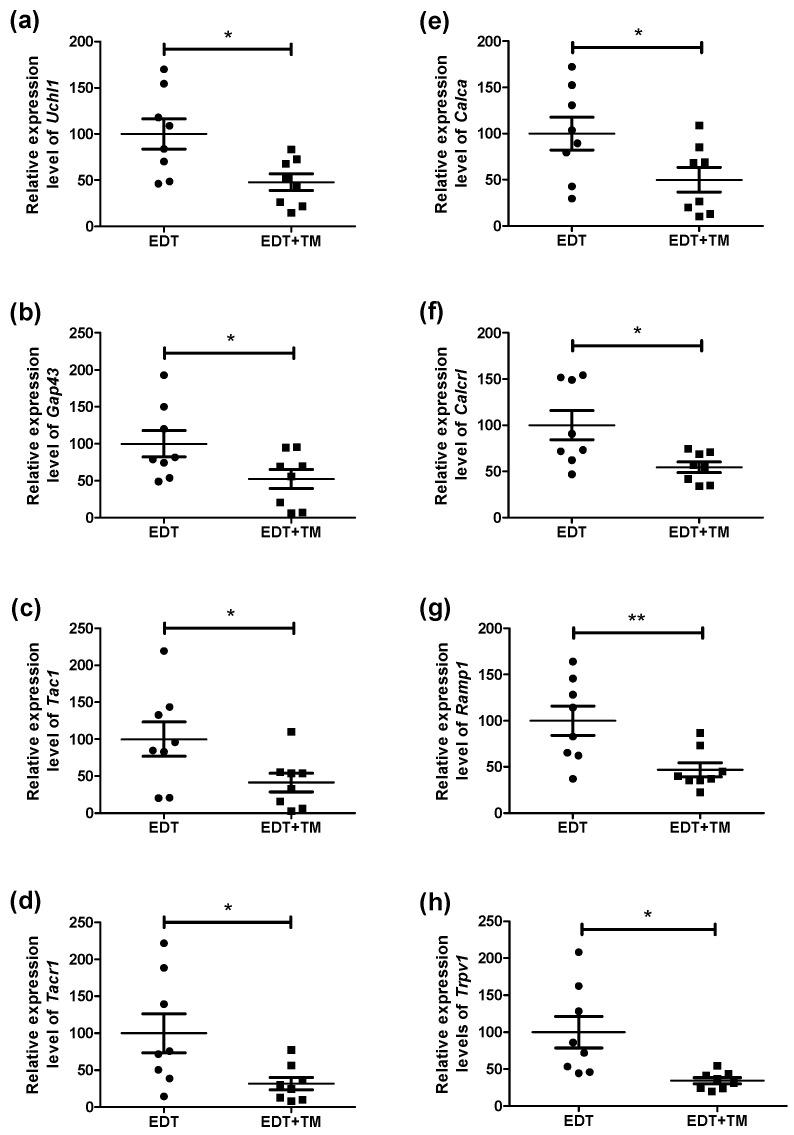
Effect of TM on the expression of neuronal and pain-related genes in endometriotic-like lesions. The mRNA expression of *Uchl1* (**a**), *Gap43* (**b**), *Tac1* (**c**), *Tacr1* (**d**), *Calca* (**e**), *Calcrl* (**f**), *Ramp1* (**g**), and *Trpv1* (**h**) was evaluated by RT-qPCR in untreated mice with induced EDT or in TM-treated mice (EDT+TM). The relative quantification of each mRNA was calculated from the Cq values obtained for the target genes and the reference gene (*Rn18s*), using the 2^−ΔΔCq^ method. Results are expressed as mean ± SEM (n = 8 animals per group). All experiments were performed in duplicate. Statistical comparisons were carried out using Student’s *t*-test (* *p* < 0.05; ** *p* < 0.01).

Since neurotrophins can stimulate the release of neuropeptides and, in turn, modulate both inflammation and pain sensitization in a dynamic and complex manner in EDT, we assessed the expression of neuronal and pain-related genes in endometriotic-like lesions. These included *Uchl1* (general neuronal marker), *Gap43* (marker of nerve fiber sprouting and growth), *Tac1*/*Tacr1* (SP signaling), *Calca*/*Calcrl*/*Ramp1* (CGRP pathway), and *Trpv1* (nociception and chronic pain). TM treatment significantly reduced the mRNA levels of all these genes compared with the EDT group (Figure 3a–h): *Uchl1* (*p* < 0.05), *Gap43* (*p* < 0.05), *Tac1* (*p* < 0.05), *Tacr1* (*p* < 0.05), *Calca* (*p* < 0.05), *Calcrl* (*p* < 0.05), *Ramp1* (*p* < 0.01), and *Trpv1* (*p* < 0.05).

### 2.3. Effect of TM on the Fibrogenesis of Endometriotic-like Lesions

**Figure 4:**
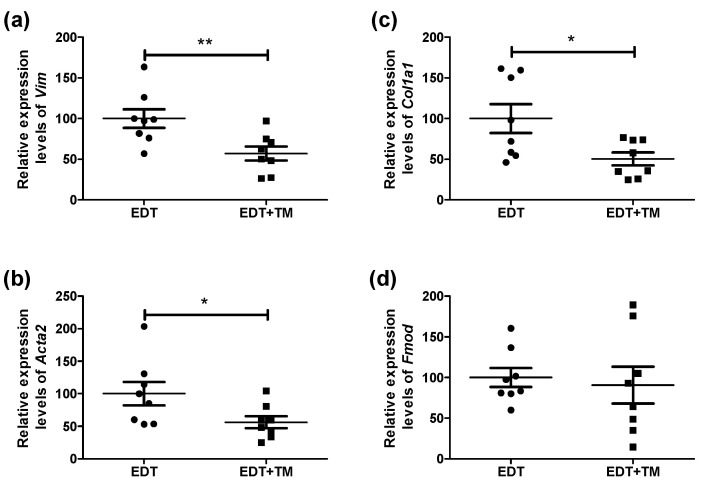
Effect of TM on the expression of fibroblast activation markers and tissue remodeling in endometriotic-like lesions. The mRNA expression of *Vim* (**a**), *Acta2* (**b**), *Col1a1* (**c**), and *Fmod* (**d**) was evaluated by RT-qPCR in untreated mice with induced EDT or in TM-treated mice (EDT+TM). The relative quantification of each mRNA was calculated from the Cq values obtained for the target genes and the reference gene (*Rn18s*), using the 2^−ΔΔCq^ method. Results are expressed as mean ± SEM (n = 8 animals per group). All experiments were performed in duplicate. Statistical comparisons were carried out using Student’s *t*-test (* *p* < 0.05; ** *p* < 0.01).

Fibrosis is a hallmark of endometriotic lesions, contributing to tissue remodeling as well as the persistence of the disease and its associated pain. Therefore, the mRNA expression of *Vim*, involved in extracellular matrix remodeling; *Acta2* and *Col1a1*, commonly used as markers of myofibroblasts and type I collagen synthesis, respectively; and *Fmod*, a proteoglycoprotein capable of binding to collagens and modulating fibrogenesis, was analyzed. Treatment with TM significantly reduced the expression of *Vim* (*p* < 0.01), *Acta2* (*p* < 0.05), and *Col1a1* (*p* < 0.05), with no changes to the *Fmod* expression, compared with the EDT group (Figure 4a–d).

**Figure 5:**
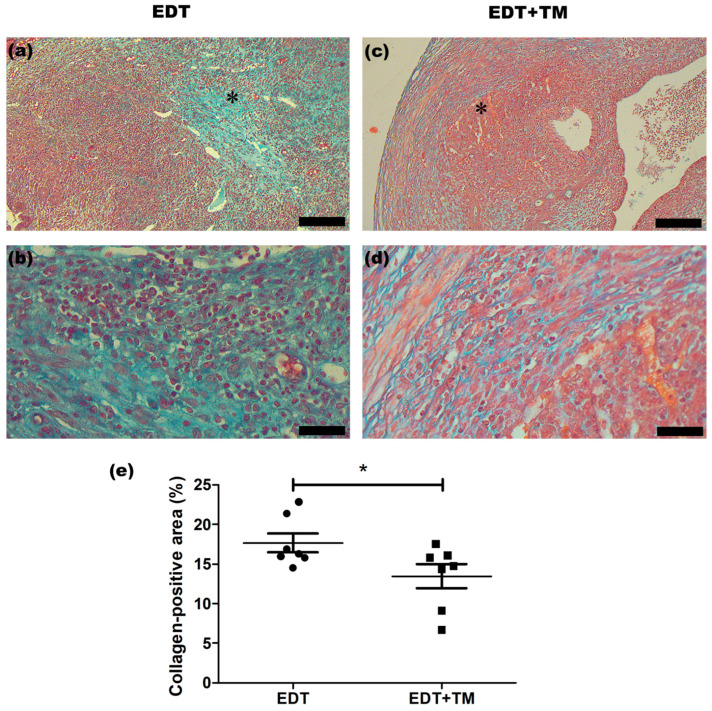
Effect of TM on collagen-positive areas in endometriotic-like lesions. Panels (**a**,**b**) show representative sections from the untreated EDT group, stained with Masson’s trichrome, at 100× and 400× magnification, respectively (scale bars: 100 μm and 25 μm). Panels (**c**,**d**) show representative sections from the TM-treated group (EDT+TM) at 100× and 400× magnification, respectively (scale bars: 100 μm and 25 μm). The asterisk in the 100× images indicates the area from which the higher-magnification micrographs were obtained. Fibrosis was quantified as the blue-green-stained area using Image-Pro Plus 6.0 and expressed as a percentage of the total tissue area (**e**). For each animal, mean values were calculated from the analyzed sections and then averaged per experimental group. Data are presented as mean ± SEM (n = 7 animals per group). Statistical comparisons were performed using Student’s *t*-test (* *p* < 0.05).

In addition, collagen content was evaluated using Masson’s trichrome staining, which revealed that collagen fibers were predominantly localized in the stromal compartment of the endometriotic-like lesions (Figure 5a–d). Quantitative analysis showed that the collagen-positive area, expressed as a percentage of the total endometriotic-like lesion area, was significantly lower in the group treated with the Cu chelator compared with the EDT control (*p* < 0.05; Figure 5e).

## 3. Discussion

**Figure 6:**
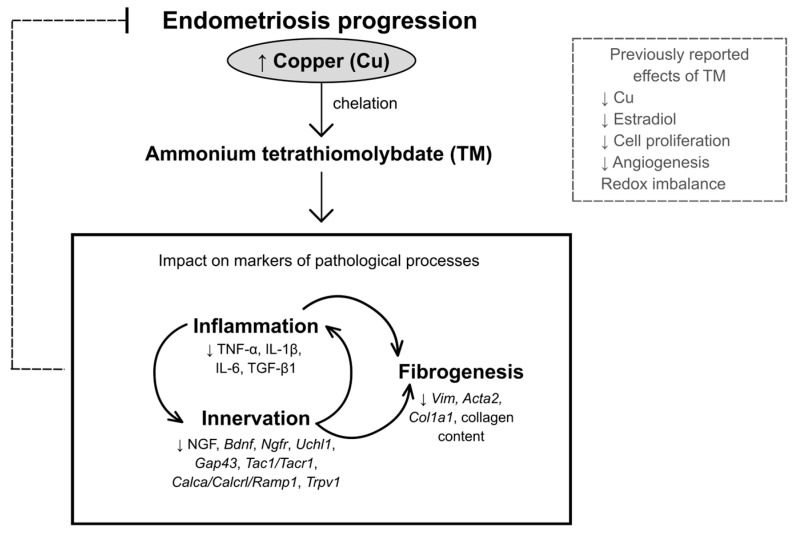
Schematic model summarizing the key findings on the effects of TM in experimental EDT. Previous studies have reported elevated Cu levels in both patients with EDT and in animal models of the disease. TM has been shown to reduce Cu and estradiol levels, inhibit cell proliferation and angiogenesis, and modulate redox imbalance. This study further demonstrates that TM modulates markers of the interconnected processes of inflammation, innervation, and fibrogenesis, collectively limiting the progression of endometriosis.

Previously, we demonstrated elevated Cu levels in the peritoneal fluid of mice with induced EDT. We also showed that TM treatment normalized Cu and estradiol levels and reduced the volume of endometriotic-like lesions by affecting cell proliferation, angiogenesis, and redox imbalance [22,23]. In the present study, we investigated how TM affects markers of inflammation, innervation, and fibrogenesis in mice with experimentally induced EDT, to assess its therapeutic potential and advance our understanding of the disease. This drug significantly altered both mRNA expression and protein levels of cytokines, as well as various neural, nociceptive, and fibrogenic markers, and reduced collagen content in endometriotic-like lesions (Figure 6). Altogether, our findings highlight TM as a promising candidate for further investigation as a potential therapy for EDT.

First, the expression of TNF-α, IL-1β, IL-6, and TGF-β1 in ectopic uterine tissue, along with their concentrations in peritoneal fluid, was evaluated in mice with and without TM treatment. These cytokines were chosen for their key role in the inflammatory processes associated with EDT. The establishment of EDT increased the cytokine concentration, mirroring the inflammatory state characteristic of the disease in humans [3,4]. Altered Cu homeostasis has been reported to drive inflammation by participating in redox reactions and promoting the production of reactive oxygen species (ROS) [15]. In addition, Cu-dependent mitochondrial signaling has been implicated in the activation of inflammatory pathways, with potential epigenetic effects on immune cells [16,18,19,21]. Therefore, Cu may indirectly activate the nuclear factor kappa B (NF-κB) signaling pathway, promoting sustained production of pro-inflammatory cytokines. This is noteworthy, as NF-κB regulates genes involved in endometriotic cell proliferation, inflammatory responses in ectopic endometrium through cytokine production, and extracellular matrix remodeling, as reported in the literature [24]. NF-κB may represent a promising target for future research into Cu-mediated effects in EDT. Furthermore, previous studies indicate that elevated levels of pro-inflammatory cytokines encourage the recruitment and activation of immune cells, including macrophages, mast cells, and neutrophils. These cells are a significant source of additional cytokines and other factors that increase the local inflammatory response. This self-perpetuating cycle of inflammation contributes to the growth of endometriotic lesions and chronic pelvic pain [3].

Conversely, TM administration produced an anti-inflammatory response, reducing the expression and concentration of TNF-α, IL-1β, IL-6, and TGF-β1 to values comparable to those in Sham animals. Previous studies on pulmonary fibrosis and cancer have demonstrated that TM can lower Cu bioavailability and suppress the expression of pro-inflammatory, pro-angiogenic, and pro-fibrotic cytokines through several mechanisms, including the NF-κB signaling pathway [25,26]. In line with these reports, the cytokine modulation observed here is consistent with TM’s effects on Cu levels, endometriotic-like lesion volume, cell proliferation, and angiogenesis in mice with induced EDT [23].

Based on the anti-inflammatory effects of TM in experimental EDT, we analyzed NGF and BDNF, neurotrophins elevated in this pathology that act as inflammatory mediators, stimulate nerve growth, and contribute to EDT-associated pain [3,6,7]. TM decreased the mRNA expression of both neurotrophins and their common receptor *Ngfr*, as well as the NGF protein levels. Although TM significantly reduced *Bdnf* mRNA expression, the decrease in BDNF protein expression did not reach statistical significance. This discrepancy could reflect the complex post-transcriptional regulation of BDNF and its compartmentalized protein dynamics [27] or suggest that the treatment’s effect may be primarily transcriptional within the analyzed experimental time window.

Cu (II), the predominant extracellular form of this metal, has been reported to modulate multiple aspects of neurotrophic signaling. These include stabilizing NGF conformation and modulating intracellular pathways regulated by neurotrophic receptors, such as the mitogen-activated protein kinase-extracellular signal-regulated kinase (MAPK-ERK) and phosphoinositide 3-kinase-protein kinase B (PI3K-AKT) pathways [28,29]. Importantly, all these proteins have been implicated in EDT development [8,9]. A possible role of Cu (II) in regulating BDNF maturation and availability has even been suggested [28,29]. Furthermore, NGF and BDNF are known to be modulated by estrogens through neuroimmune interactions, which may affect the expression of their common receptor [7,30]. Thus, the decrease in Cu bioavailability induced by TM in our experimental model, along with reduced estradiol levels [23], could explain the observed decreases in NGF, *Bdnf*, and *Ngfr*.

Importantly, NGF stimulates the expression of CGRP and SP in sensory neurons and sprouting and sensitization of peripheral sensory nerve fibers, thereby enhancing nociceptor activation. In turn, BDNF promotes the growth, differentiation, and plasticity of several neuronal populations [7]. Therefore, the reduction in neurotrophins observed in TM-treated animals could explain the decreased expression of neuronal markers, neuropeptides, and their receptors. This reduction is also linked to the anti-inflammatory effect observed in animals treated with the Cu chelator. Inflammation around endometriotic lesions is known to sensitize nerve fibers, promoting the release of neuropeptides. Both CGRP and SP are pro-inflammatory neuropeptides that increase vasodilation and vascular permeability, recruit immune cells, and promote the release of pro-inflammatory cytokines [7]. Taken together, and given the strong interconnection between inflammation and innervation, it is not surprising that TM simultaneously affected markers of both processes, which are fundamental to the EDT progression. These findings suggest that TM may attenuate pain-associated neurotrophin- and neuropeptide-driven sensitization mechanisms. Consistently, a decrease in *Trpv1* mRNA expression was observed, which could have implications for pain signaling. TRPV1 is upregulated in endometriotic tissues, and its increased expression has been associated with greater pain intensity. This channel contributes to peripheral sensory fiber sensitization and the modulation of inflammatory pathways, amplifying nociception [31,32]. Thus, a reduction in its expression due to TM treatment would align with the observed decrease in cytokine expression and may contribute to reduced nociceptive activation.

Fibrosis, sustained by the interaction between inflammation and aberrant innervation, is another key therapeutic target in this complex disease [8,9,12]. In this study, TM decreased the expression of *Vim*, *Acta2*, and *Col1a1* in endometriotic-like lesions, while *Fmod* remained unchanged. These findings suggest that TM could interfere with the transition to a mesenchymal phenotype and myofibroblast differentiation, which would limit the acquisition of smooth muscle cell-like characteristics and hinder collagen secretion. Indeed, TM-treated endometriotic-like lesions showed a lower percentage of collagen-positive areas, consistent with the decreased TGF-β, a recognized driver of pathological fibrosis. This observation aligns with the reduced expression of innervation markers in TM-treated animals, as higher TGF-β1 levels have been reported in nerve fibers of peritoneal endometriotic lesions compared to non-endometriotic peritoneum [9]. Additionally, the reduced expression of the analyzed neuropeptides is consistent with the antifibrotic effects of TM. Previous evidence shows that CGRP and SP promote EMT, fibroblast-to-myofibroblast transdifferentiation, and the transformation of stromal cells into smooth muscle cells in endometriotic lesions. Specifically, fibrotic endometriotic lesions were characterized by increased vimentin-positive stromal cells, accumulation of myofibroblasts expressing α-SMA (encoded by the *Acta2* gene in mice), and excessive collagen deposition, highlighting the contribution of EMT and fibroblast activation to lesion fibrogenesis [33]. Furthermore, Cu has also been implicated in the fibrotic process in multiple pathological contexts, acting through both lysyl oxidase-dependent matrix crosslinking and ROS-mediated EMT-related mechanisms [13,34,35]. In animal models of fibrosis, TM exerted antifibrotic effects [25,35,36] by attenuating TGF-β–driven EMT, reducing collagen I expression, restoring E-cadherin levels, and inhibiting the Cu transporter proteins. It is conceivable that similar mechanisms may contribute to fibrogenesis in EDT and are being affected by TM, as suggested by the observed changes in fibrogenic markers, warranting further investigation. Interestingly, Anchan and colleagues [37] established the C57BL/6J strain as a suitable model for studying EDT, consistently recapitulating the inflammatory and fibrotic pathophysiology observed in humans. This similarity between the human disease and the experimental model is particularly promising, as fibrosis contributes to symptom severity and the persistence of endometriotic lesions [8,9]. In this context, the putative antifibrotic effects of TM observed in this model, combined with its anti-inflammatory actions and modulation of innervation- and nociception-related markers, further highlight the interconnection among these processes and suggest a therapeutic relevance in EDT.

In summary, our findings indicate that Cu chelation with TM attenuates key pathological processes in EDT, such as inflammation, aberrant innervation, and fibrogenesis. TM restored the analyzed cytokine profile, reduced neurotrophins and neuropeptide signaling associated with neuronal sensitization, and attenuated fibroblast activation markers, tissue remodeling markers, and collagen-positive area. These results, alongside previously reported effects of TM on cell proliferation, angiogenesis, and redox imbalance [22,23], reinforce the notion that Cu availability plays a critical role in EDT progression. They also suggest that TM may represent a potential approach to modulate multiple interconnected pathological mechanisms in EDT. In addition to its favorable safety profile, TM (or similar Cu chelators) offers a non-hormonal treatment approach that could help overcome some of the drawbacks of current EDT therapies. These issues include limited effectiveness in stopping endometriotic lesion growth, side effects of long-term hormonal therapy, disease recurrence after treatment or surgery, and risks from invasive procedures [1,2]. A limitation of this study is that the murine EDT model is induced, as rodents do not spontaneously develop the disease. It means it only partially represents the complex and multifactorial nature of EDT in humans. Despite this, the experimental model is widely accepted for preclinical evaluation of possible therapies, offering significant ethical and practical advantages over non-human primate models. Further investigations are needed to clarify the molecular mechanisms underlying altered Cu homeostasis and metal chelation in EDT, as they may provide a robust biological basis for the rational design of future translational and clinical research.

## 4. Materials and Methods

### 4.1. Animal Handling

Two-month-old female wild-type C57BL/6 mice (*Mus musculus*), weighing 19–21 g, were used in this study. Breeding colonies were established in the Animal Facility of the Universidad Nacional de San Luis (San Luis, Argentina). The animal room followed strict lighting conditions (12 h light/12 h dark cycle) and controlled temperature (22 ± 2 °C), with ad libitum access to sterile water and food. All experimental procedures complied with the Guide for the Care and Use of Laboratory Animals of the National Research Council [38] and complied with the ARRIVE guidelines 2.0.

### 4.2. Induced EDT Through Surgical Procedures in Mice

Twenty-four mice were randomly assigned to three experimental groups (n = 8 animals per group): (1) Sham (sham-operated animals), (2) EDT (animals with induced disease), and (3) EDT+TM (animals with disease treated with TM). EDT was induced by autologous transplantation of 4 mm^2^ uterine tissue fragments into the intestinal mesentery, sutured with Ethicon 6-0 (Somerville, NJ, USA). In sham-operated mice, three tissue-free sutures were placed. Surgeries were performed under anesthesia using a ketamine (100 mg/kg; Holliday Scott, Buenos Aires, Argentina) and xylazine (10 mg/kg; Richmond, Buenos Aires, Argentina) mixture. The general health of the animals was monitored daily by recording body weight, food intake, and grooming behavior. One month after surgery, animals were euthanized by cervical dislocation. The peritoneal cavity was immediately lavaged with 1.5 mL of phosphate-buffered saline (PBS; pH 7.4) per animal. The lavage fluid was centrifuged at 250× *g* for 10 min at 4 °C, and the supernatant (peritoneal fluid) was stored at −80 °C until further analysis. Finally, the abdomen was completely opened to access endometriotic-like lesions. For each animal, one endometriotic-like lesion was fixed in 4% paraformaldehyde in PBS (pH 7.4) for 24 h at 4 °C. Fixed samples were embedded in paraffin and cut into 4-μm-thick sections. Standard hematoxylin–eosin staining confirmed the presence of endometrial glands and stroma in ectopic tissue. Additional 4-μm sections were prepared for Masson’s trichrome staining. Another endometriotic-like lesion was placed at −20 °C in RNAhold^®^ (TransGen Biotech^®^ Co., Ltd., Beijing, China) for RT-qPCR studies. The third endometriotic-like lesion was kept at −80 °C for protein extraction.

### 4.3. TM Administration

Starting on postoperative day 15, after the uterine tissue had established itself at the ectopic sites, each animal in group 3 received 0.3 mg of TM (Cat. #323446, Sigma-Aldrich, St. Louis, MO, USA) orally, as described by Delsouc and colleagues [23]. This dose reduces Cu levels to values comparable to those of sham mice, without affecting their overall well-being, and maintains hematocrit above 80% of the baseline value [22,23].

### 4.4. Reverse Transcription–Quantitative Polymerase Chain Reaction (RT-qPCR)

RT-qPCR was performed to analyze the expression of the following genes: *Tnfa*; *Il1b*; *Il6*; *Tgfb1*; *Ngf*; *Bdnf*; NGF receptor (*Ngfr*); ubiquitin carboxy-terminal hydrolase-L1 (*Uchl1*); growth-associated protein 43 (*Gap43*); tachykinin precursor 1 (*Tac1*); tachykinin receptor 1 (*Tacr1*); calcitonin/calcitonin-related polypeptide alpha (*Calca*); calcitonin receptor-like (*Calcrl*); receptor activity-modifying protein 1 (*Ramp1*); transient receptor potential cation channel subfamily V member 1 (*Trpv1*); vimentin (*Vim*); alpha-smooth muscle actin (*Acta2*); collagen type I alpha 1 (*Col1a1*); and fibromodulin (*Fmod*). Total RNA was isolated from endometriotic-like lesions using TRIzol^®^ reagent (Thermo Fisher Scientific, Inc., Waltham, MA, USA). The RNA concentration was determined with an EPOCH™ microplate spectrophotometer (BioTek Instruments, Inc., Winooski, VT, USA). All RNA samples were treated with RQ1 RNase-Free DNase (Promega Co., Madison, WI, USA). Subsequently, 1 μg of total RNA was converted into cDNA using the Transcriptor First Strand cDNA Synthesis Kit (Roche Diagnostics International, Ltd., Mannheim, Germany), following the manufacturer’s protocol, and stored at −20 °C. Quantitative PCR (qPCR) was conducted using an ABI PRISM^®^ 7500 Instrument (Applied Biosystems^®^, Waltham, MA, USA) and the FastStart™ Universal SYBR^®^ Green Master (Roche Diagnostics International, Ltd., Mannheim, Germany). The reaction mixture consisted of 2 × FastStart™ Universal SYBR^®^ Green Master Mix, cDNA, forward primer (10 μM), reverse primer (10 μM), and nuclease-free water. Primer sequences are listed in Table 1 (Integrated DNA Technologies, Inc., Coralville, IA, USA). PCR cycling conditions were an initial denaturation at 95 °C for 10 min, followed by 40 cycles of 95 °C for 15 s and 60 °C for 1 min. The relative expression was calculated using the 2^−ΔΔCq^ method. All experiments were performed in duplicate, and *Rn18s* (18S ribosomal RNA) was used as the internal reference gene.

### 4.5. Enzyme-Linked Immunosorbent Assay (ELISA)

The protein levels of the cytokines TNF-α, IL-1β, IL-6, and TGF-β1 were analyzed in peritoneal fluid samples using the following commercial kits, in accordance with the manufacturer’s protocols: Mouse TNF-alpha DuoSet ELISA (Cat. #DY410, R&D Systems, Minneapolis, MN, USA); ELISA MAX™ Deluxe Set Mouse IL-1β (Cat. #432604, BioLegend, Inc., San Diego, CA, USA); Mouse IL-6 Uncoated ELISA Kit (Cat. #88-7064-88, Invitrogen, Vienna, Austria); Mouse TGF-beta 1 DuoSet ELISA (Cat. # DY1679, R&D Systems, Minneapolis, MN, USA). In addition, NGF and BDNF expression were analyzed in endometriotic-like lesion homogenate supernatants obtained with radioimmunoprecipitation assay (RIPA) buffer (Thermo Fisher Scientific, Inc., Waltham, MA, USA). The suspensions were centrifuged at 21,912× *g* for 15 min at 4 °C to remove nuclei and cell debris. The total protein concentration was determined by the Bradford method [39]. The ELISA technique was performed on 96-well microplates (Corning Incorporated, Corning, NY, USA). To 20 µg of total protein, 180 µL of 0.1 M bicarbonate buffer (pH 9.6) was added and incubated for 1 h at 37 °C. The plates were then washed with PBS containing 0.05% Tween 20 (PBS-T) and blocked with 5% skim milk in PBS-T for 1 h at 37 °C. Subsequently, the microplates were incubated with 50 µL of the primary antibody solution (diluted 1:1000 in 1% blocking buffer) at 4 °C overnight with agitation. The primary antibodies used were mouse anti-NGF (sc-365944, Santa Cruz Biotechnology, Inc., Dallas, TX, USA) and rabbit anti-BDNF (sc-20981, Santa Cruz Biotechnology, Inc., Dallas, TX,, USA). After five successive washes with PBS-T, 50 µL of a solution containing the secondary antibody was added to each well: anti-mouse IgG (dilution 1:10,000, Jackson Immuno-Research Labs, West Grove, PA, USA) or anti-rabbit IgG (dilution 1:5000, sc-2005, Santa Cruz Biotechnology, Inc., Dallas, TX, USA), respectively, conjugated with horseradish peroxidase, and incubated for 1 h at 37 °C. Finally, the immune complexes were quantified using 3,3′,5,5′-tetramethylbenzidine (TMB). The substrate oxidation reaction was stopped with 2 M sulfuric acid, and the optical density (OD) was measured at 450 nm using an EPOCH microplate reader (BioTek Instruments, Inc., Winooski, VT, USA).

### 4.6. Masson’s Trichrome Staining

Staining was performed to assess collagen content in endometriotic-like lesions, with collagen fibers visualized in blue-green, nuclei in dark blue, and muscle/cytoplasm in red. Slides were examined with an Olympus BX-50 microscope equipped with a digital camera (MShot, MS60). The images were processed using Image-Pro Plus 6.0 (Media Cybernetics, Inc., Rockville, MD, USA). For each section, the collagen-positive area and total tissue area were quantified, and the collagen-positive area was expressed as a percentage of total tissue area. Mean values were calculated per animal and for each experimental group.

### 4.7. Statistical Analysis

Statistical analysis was conducted using GraphPad Prism 5.0 software (GraphPad Software, Inc., San Diego, CA, USA). Values were presented as the mean ± SEM (standard error of the mean). Comparisons between two groups were performed using a two-tailed unpaired Student’s *t*-test; when the assumption of equal variances was not met (F-test, *p* < 0.05), Welch’s correction was applied. For multiple group comparisons, one-way ANOVA followed by Tukey’s post hoc test was used. A *p*-value < 0.05 was considered statistically significant.

## Figures and Tables

**Table 1:** Primer gene symbols, sequences, GenBank access numbers, and sizes of amplicons.

Gene	Sequences (5′–3′)	GenBank Access Number	Amplicon
*Tnfa*	Forward: CTACCTTGTTGCCTCCTCTTT Reverse: GAGCAGAGGTTCAGTGATGTAG	NM_013693.3	116 bp
*Il1b*	Forward: CTTCCAGGATGAGGACATGAG Reverse: TCACACACCAGCAGGTTATC	NM_008361.4	102 bp
*Il6*	Forward: GTCTGTAGCTCATTCTGCTCTG Reverse: GAAGGCAACTGGATGGAAGT	NM_031168.2	102 bp
*Tgfb1*	Forward: CTGAACCAAGGAGACGGAATAC Reverse: GGGCTGATCCCGTTGATTT	NM_011577.2	101 bp
*Ngf*	Forward: AGTGTCTGGGCCCAATAAAG Reverse: CGCAGTGATCAGAGTGTAGAAC	NM_013609.3	106 bp
*Bdnf*	Forward: CTGAGCGTGTGTGACAGTATTA Reverse: CTTTGGATACCGGGACTTTCTC	NM_007540.4	112 bp
*Ngfr*	Forward: CATTCCTGTCTATTGCTCCATCT Reverse: GCTGTTGGCTCCTTGTTTATTT	NM_033217.3	109 bp
*Uchl1*	Forward: CAACCAAGACAAGCTGGAATTT Reverse: TCTCGAAACACTTGGCTCTATC	NM_011670.2	101 bp
*Gap43*	Forward: GTGCTGTATGAGAAGAACCAAAC Reverse: GCAGCCTTATGAGCCTTATCT	NM_008083.2	99 bp
*Tac1*	Forward: CATGGCCAGATCTCTCACAAA Reverse: GCATCGCGCTTCTTTCATAAG	NM_009311.3	100 bp
*Tacr1*	Forward: GACCGTTACCATGAGCAAGT Reverse: CAGGAGGAAGAAGATGTGGAAG	NM_009313.5	111 bp
*Calca*	Forward: CCCCAGAATGAAGGTTACACA Reverse: TGTCAAAGGGAGAAGGGTTTT	NM_001033954	138 bp
*Calcrl*	Forward: GTCCGATTTGTGCTGCTTTG Reverse: GGATTCCACTTGGTGTGTAACT	NM_018782.2	98 bp
*Ramp1*	Forward: GCTGGCTCACCATCTCTTC Reverse: CCCAATAGTCTCCATGTTCTCC	NM_016894.3	109 bp
*Trpv1*	Forward: GAGACCTGTGTCGGTTTATGT Reverse: CTCCACAGGCAGTGAGTTATT	NM_001001445.2	107 bp
*Vim*	Forward: CATTGAGATCGCCACCTACAG Reverse: TCTCTCAGGTTCAGGGAAGAA	NM_011701.4	93 bp
*Acta2*	Forward: CAGCCATCTTTCATTGGGATG Reverse: ACAGGACGTTGTTAGCATAGAGA	NM_007392.3	112 bp
*Col1a1*	Forward: GCCAAGAAGACATCCCTGAA Reverse: TCAAGCATACCTCGGGTTTC	NM_007742.4	87 bp
*Fmod*	Forward: CTCTGCCACATTCTCCAACC Reverse: CACTGCATTTTTGTCTCTTGG	NM_021355.4	86 bp
*Rn18s*	Forward: CTGAGAAACGGCTACCACATC Reverse: GCCTCGAAAGAGTCCTGTATTG	NR_003278.3	107 bp

## Data Availability

The original contributions presented in this study are included in the article. Further inquiries can be directed to the corresponding author.

## Abbreviations

The following abbreviations are used in this manuscript:

*Acta2*	Alpha-smooth muscle actin
AKT	Protein kinase B
BDNF	Brain-derived neurotrophic factor
*Calca*	Calcitonin/calcitonin-related polypeptide alpha
*Calcrl*	Calcitonin receptor-like
CGRP	Calcitonin gene-related peptide
*Col1a1*	Collagen type I alpha 1
Cu	Copper
EDT	Endometriosis
ELISA	Enzyme-linked immunosorbent assay
EMT	Epithelial–mesenchymal transition
ERK	Extracellular signal-regulated kinase
*Fmod*	Fibromodulin
*Gap43*	Growth-associated protein 43
IL	Interleukin
MAPK	Mitogen-activated protein kinase
NF-κB	Nuclear factor kappa B
NGF	Nerve growth factor
*Ngfr*	NGF receptor
OD	Optical density
PBS	Phosphate-buffered saline
PI3K	Phosphoinositide 3-kinase
*Ramp1*	Receptor activity-modifying protein 1
RIPA	Radioimmunoprecipitation assay buffer
*Rn18s*	18S ribosomal RNA
ROS	Reactive oxygen species
RT-qPCR	Reverse transcription–quantitative polymerase chain reaction
SEM	Standard error of the mean
SP	Substance P
*Tac1*	Tachykinin precursor 1
*Tacr1*	Tachykinin receptor 1
TGF-β	Transforming growth factor beta
TM	Ammonium tetrathiomolybdate
TMB	3,3′,5,5′-tetramethylbenzidine
TNF	Tumor necrosis factor
*Trpv1*	Transient receptor potential cation channel subfamily V member 1
*Uchl1*	Ubiquitin carboxy-terminal hydrolase-L1
*Vim*	Vimentin
